# Selection of a Fermentation Strategy for the Preparation of Clam Sauce with Acceptable Flavor Perception

**DOI:** 10.3390/foods12101983

**Published:** 2023-05-13

**Authors:** Tao Zhou, Yunjiao Ma, Wei Jiang, Baoshang Fu, Xianbing Xu

**Affiliations:** National Engineering Research Center of Seafood, Collaborative Innovation Center of Provincial and Ministerial Co-Construction for Seafood Deep Processing, School of Food Science and Technology, Dalian Polytechnic University, Dalian 116034, China; tt20220214@163.com (T.Z.); mayunjiao1028@163.com (Y.M.); 15640852258@163.com (W.J.); fubaoshang@dlpu.edu.cn (B.F.)

**Keywords:** clam sauce, flavor, correlation analysis, free amino acid, HS-SPME-GC-MS

## Abstract

Flavor, which mainly depends on volatile compounds, is an important index for evaluating the quality of clam sauce. This study investigated the volatile compounds in clam sauce prepared using four different methods and the influence of aroma characteristics. Fermenting a mixture of soybean koji and clam meat improved the flavor of the final product. Solid-phase microextraction (SPME) combined with gas chromatography-mass spectrometry (GC-MS) identified 64 volatile compounds. Nine key flavor compounds, namely, 3-methylthio-1-propanol, 2-methoxy-4-vinylphenol, phenylethyl alcohol, 1-octen-3-ol, α-methylene phenylacetaldehyde, phenyl-oxirane, 3-phenylfuran, phenylacetaldehyde, and 3-octenone, were selected using variable importance in projection (VIP). The results of the electronic nose and tongue detection of the aroma characteristics of the samples prepared by four different fermentation methods were consistent with those of GC-MS analysis. The clam sauce prepared by mixing soybean koji with fresh clam meat possessed better flavor and quality than that prepared via other methods.

## 1. Introduction

Condiments could coordinate the smell and taste of food, increase flavor and remove the fishy smell [1]. In 2022, the scale of the condiment market in China reached CNY 513.3 billion, and the total annual output exceeded 13 million tons. There is a broad market for the condiment industry [2]. With people’s increasing demand for nutrition, naturalness and diversity of condiments, fermented seasonings are widely selected in cooking or food preparation because of their delicious taste, rich aroma and unique flavor [3]. The seafood flavor condiment with excellent umami taste is especially popular within the condiment market [4]. One of the most cultured seafoods is clam, the annual output of which reached about 4.28 million tons per year, with an annual output value of USD 150 million [5]. In addition, clam meat is rich in protein (9.09%~12.75%) [6], which has potential for the preparation of the fermented condiment.

At present, there are few reports about the preparation of clam fermented sauce. Nevertheless, similar to the preparation of fish sauce, clam meat could be fermented at high salt concentrations for sauce preparation [7]. The fermentation cycle of a traditional sauce production process requires at least six months [8]. The fermentation time is shortened by increasing the fermentation temperature and adding high-concentration saltwater to inhibit the growth of bacteria [9]; however, the salinity of clam sauce produced by this method is exceedingly high, which affects its taste. Flavor formation in fermentation sauce is a complex process [10] in which the proteins in raw meat are degraded into taste compounds such as amino acids or peptides through biochemical metabolic pathways, under the joint action of microorganisms and enzymes [11]. In addition to these flavor precursor substances, such as amino acids, fatty acids could further produce a variety of small-molecule flavor compounds such as aldehydes, ketones, alcohols, and pyrazines, which ultimately contribute to the flavor and quality of clam sauce products [12]. To some extent, different fermentation strategies will produce different flavor precursors and flavor compounds, resulting in a different flavor of the final product. Therefore, it is necessary to select a fermentation strategy to prepare clam sauce with acceptable flavor.

This study identified volatile compounds in clam sauce using headspace solid-phase microextraction combined with gas chromatography-mass spectrometry (HS-SPME-GC-MS), and further analyzed the volatile compounds in clam sauces produced by different fermentation methods using heatmap and partial least squares discriminant analyses (PLS-DA). In addition, the sensory values measured by an electronic nose and tongue were selected as the key evaluation indexes, along with the contents of free amino acids and amino acid nitrogen (AAN). Finally, the optimum fermentation method was selected by comparison of the observed volatile flavor substances, which provided a theoretical reference for the preparation of high-quality clam sauce products.

## 2. Materials and Methods

### 2.1. Materials

Trichloroacetic acid (TCA, 99%) was purchased from Macklin (Shanghai, China). Acetone (99.5%) was purchased from Damao Chemical Reagent Co., Ltd. (Tianjin, China). Hydrochloric acid (HCl, AR) was purchased from Xilong Chemical (Shantou, China). *Ruditapes philippinarum* and soybeans were purchased from the local seafood market and a local supermarket, respectively, in Dalian, China. All other reagents used in this study were of analytical grade or higher purity.

### 2.2. Preparation of Bean Koji

Soybeans were screened, cleaned, and soaked for 8–10 h at 25 °C, based on the standard degree of the soaking of soybeans covered with water. The soybeans were drained and cooked in a sterilized pot (100 °C) for 2 h [13]. After cooking, the soybeans were cooled to approximately 40 °C before adding *Aspergillus oryzae* and flour (*w*/*w* 1:60). The mixture was then covered with two layers of clean, wet gauze and cultured at 31 °C and 85% humidity for 2–3 days.

### 2.3. Preparation of Clam Meat Koji

Clam meat was used in this study. The clam meat was washed with water 2–3 times to remove impurities and then broken up before being mixed with flour (85% clam meat, 15% flour) and cooked in a sterile pot (100 °C) for 1 h. When the temperature dropped to approximately 40 °C, the clam meat was cut into small pieces (2.5 cm × 2.5 cm × 1.4 cm). The clam meat was then mixed with *Aspergillus oryzae* and flour at a ratio of 1:60 (*w*/*w*), stirred well, and covered with two layers of clean, wet gauze and incubated at 31 °C and 85% humidity for culturing [13].

### 2.4. Preparation of Mixed Koji

The soybeans were screened, cleaned, and soaked for 8–10 h at room temperature, based on the standard degree of the soaking of soybeans covered with water. High-quality clam meat was washed with water 2–3 times to remove impurities and then broken up. Soybeans (55%) and clam meat (30%) were then mixed with flour (15%) and cooked in a sterile pot (100 °C) for 1 h. When the temperature dropped to approximately 40 °C, the clam meat was cut into small pieces (2.5 cm × 2.5 cm × 1.4 cm) and mixed with *Aspergillus oryzae* and flour in a ratio of 1:60 (*w*/*w*). The mixture was stirred well, covered with two layers of clean, wet gauze and incubated at 31 °C and 85% humidity for culturing [14].

### 2.5. Preparation of Fermented Clam Sauce

Various fermentation methods were used to prepare the clam sauce. The precursor of sauce I was mixed koji (100%). The precursors of sauce II were meat koji (67%) and soybean koji (33%). The precursors of sauce III were clam meat (67%) and soybean koji (33%). The precursor of sauce IV was meat koji (100%).

The precursor of each sauce was mixed with salt water (12%) and fermented at 45 °C for 12 days, then at 35 ℃ for 18 days. The mixture was stirred every two days during the fermentation process to produce a delicious and fragrant clam sauce [15], which was stored at −30 °C prior to use.

### 2.6. E-Tongue Analysis

The taste of the clam sauce was analyzed using a TS-5000Z electronic tongue (Insent Inc., Japan), employing an established method with some modifications [16]. The sensors on the electronic tongue included bitterness (SB2C00), umami (SB2AAE), salinity (SB2CT0), acidity (SB2CA0), and astringency sensors (SB2AE1). A sample of the clam sauce (2 g) was added to deionized water (100 mL) and shaken well. After centrifugation, the supernatant (60 mL) was collected for testing. Each sample was analyzed using three parallel experiments.

### 2.7. Amino Acid Nitrogen Analysis

The reference analysis of the AAN content in soy sauce was conducted in accordance with the official analytical method in China (GB/T 5009.235—2016). The AAN content was determined using the titration method, in which diluted samples (20 mL) were mixed with distilled water (60 mL) and titrated to pH 8.2 with 0.05 mol/L NaOH (0.05 mol/L). Formaldehyde solution (10 mL, 40%) was then added, and the mixture was titrated to pH 9.2 with NaOH (0.05 mol/L). The AAN content was calculated from the volume of NaOH and a blank test was performed using distilled water [17].

### 2.8. Amino Acid Analysis

The amino acids in the clam sauces were identified using a Hitachi LA8080 amino acid analyzer (LA8080, Hitachi, Japan) with a high-performance appraisal-exchange column (Hitachi, Japan) [18]. Clam sauces (0.1 g) were diluted 10 times in distilled water before being mixed with an equal volume of TCA (10%). The mixture was maintained at 4 °C for 1 h to precipitate the protein and then centrifuged at 10,000× *g* for 10 min. The supernatant was collected, and the centrifugation procedure was repeated. Hydrochloric acid was added to obtain a final hydrochloric acid concentration of 0.02 M and the sample was passed through a filter (0.22 µm). The samples were eluted at 57 °C in the column oven, and the derivatization of amino acid with ninhydrin was performed at 135 °C in the reaction oven. The injection volume was 20 µL.

### 2.9. E-Nose Analysis

The flavor profile of the clam sauce was analyzed using the PEN3 electronic nose (WinMuster Airsense Analytics Inc., Schwerin, Germany), which contains 10 metal oxide sensors that have different sensitivities for each characteristic volatile compound [19]. The substances to which each type of sensing element is sensitive are listed in Table 1. The samples of the clam sauce (5.0 g) were shaken vigorously in an electronic nose (e-nose) sample bottle (20 mL). After cleaning, the probe with filtered air, the inlet of the electronic nose was inserted into the sample bottle and analysis was performed over 100 s. After analysis, the probe was cleaned with filtered air for 20 s [20].

### 2.10. GC-MS Analysis

The characteristic flavor compounds of the clam sauce were identified using HS-SPME-GC-MS. The clam sauce (0.5 g) was added to a cyclohexanone internal standard (10 mg/L) in a headspace bottle (10 mL) before extraction. The volatile compounds were extracted in headspace vials and incubated in a water bath at 60 °C for 20 min. The lab-made SPME fiber with a hydrophilic–lipophilic balance (HLB) [21] was exposed to the headspace for 40 min. The fibers were then injected into the gas chromatography (GC) system, which operated at 250 °C for 10 min in the splitless mode to thermally desorb the extracted compounds [22]. 

GC was performed using an Agilent 7890 B chromatograph (Agilent Technologies Inc., Santa Clara, CA, USA) with a non-polar HP-5 ms ultra-inert Agilent column (30 m × 250 µm × 0.25 µm). The initial temperature of the GC oven was 35 °C, which was increased to 230 °C at a rate of 5 °C/min and maintained for 10 min. Helium was used as the carrier gas at a flow rate of 1 mL/min. MS (5977 B, Agilent Technologies Inc., Santa Clara, CA, USA) detection was performed in scan mode, within a scanning range of m/z 35–500, and used an electron impact ion source (70 eV) at 230 °C [23].

### 2.11. Statistical Analysis

All experiments were conducted in triplicate, and the results are presented as the mean and standard deviation of the three experiments. The SPSS software package (SPSS 10.0 for Windows) was used to determine significant differences among the experiments (*p* < 0.05), using analysis of variance (ANOVA).

## 3. Results and Discussion

### 3.1. Analysis of the Taste of Clam Sauces by an Electronic Tongue

**Figure 1:**
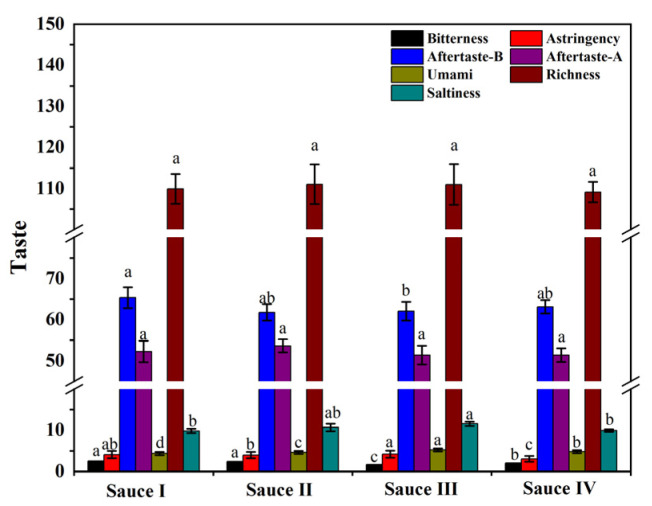
Electronic tongue (E-tongue) analysis of clam sauces prepared using different fermentation methods (sauce I, sauce II, sauce III, and sauce IV). Different lowercase letters indicate significant differences in the average value within each group (*p* < 0.05).

The electronic tongue, using an artificial lipid membrane sensor, was used to quantify the six basic tastes of clam sauces, including taste, bitterness, astringency, richness, aftertaste astringency (aftertaste-A), and aftertaste bitterness (aftertaste-B) [24]. The saltiness response values of the clam sauces prepared by the four different processes were all high (Figure 1), which may occur because clams are mariculture species with a strong capacity to adsorb substances from the environment. In addition, the preparation of clam sauce requires extra salt, further increasing its saltiness. In contrast, sauces I, II, III, and IV were all less bitter, likely because the raw clam proteins in sauce III were hydrolyzed into small peptides and had less exposure to hydrophobic amino acid residues [25]. The umami response values of sauce III were significantly higher than those of the other three sauces. These results demonstrate that sauce III had a better taste than sauces I, II, and IV.

### 3.2. Analysis of Amino Acid Nitrogen in Clam Sauces

**Figure 2:**
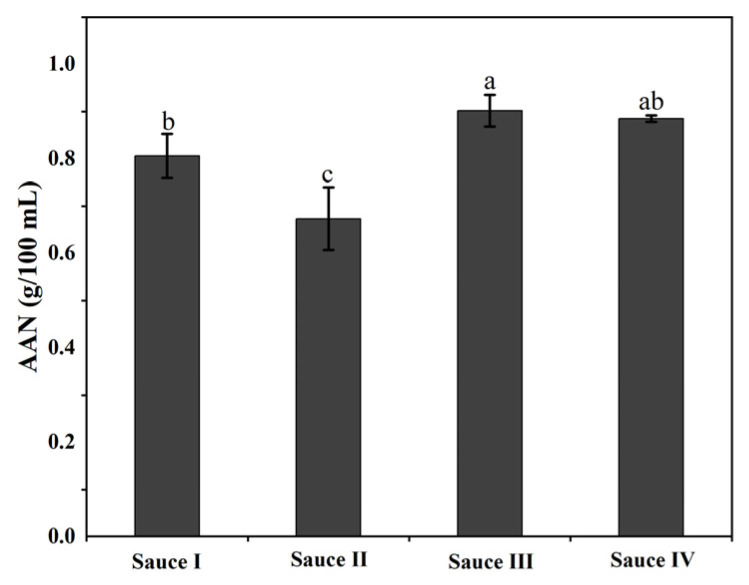
Amino acid nitrogen (AAN) analysis of the clam sauces prepared using different fermentation methods (sauce I, sauce II, sauce III, and sauce IV). Different lowercase letters indicate significant differences in the average value within each group (*p* < 0.05).

The free amino acid content of clam sauces is mainly characterized by the content of amino acid nitrogen, which reflects the fermentation maturity and flavor characteristics of the clam sauce [26]. The amino acid content is an important component of the umami substance, that is crucial in forming the flavor of the clam sauce [27]. The amino acid nitrogen contents of the clam sauces are shown in Figure 2, in which sauce I, sauce II, sauce III, and sauce IV are all higher than the Chinese national standard of 0.5 g/100 mL, and the product therefore satisfies the quality requirements of this standard. The amino acid nitrogen content in sauce III was higher than that of the other three clam sauces (*p* < 0.05), indicating that sauce III had the strongest umami flavor and good product quality. This is consistent with the electronic tongue analysis of the sauces.

### 3.3. Analysis of Free Amino Acids in Clam Sauces

The main taste substances in clam sauce are amino acids, which play an important role in the formation of flavors [28] and are released during the degradation of proteins in clam meat. A total of 14 free amino acids were detected in the sauce samples produced by the four fermentation processes (Table 2). The contents of total free amino acids (TFAA) in clam sauce I, sauce II, sauce III, and sauce IV were 2193.84 mg/100 g, 2016.22 mg/100 g, 2343.69 mg/100 g, and 2172.89 mg/100 g, respectively. The TFAA content of sauce III was significantly higher than that of the other three sauces (*p* < 0.05), likely owing to the more complete degradation of the protein during the fermentation process. The increased amino acid content of sauce III facilitates its digestion and absorption. The main delicious amino acids (FAAs) in clam sauce are Glu, Asp, Gly, and Ala. The total content of these umami amino acids is higher than 30%, which may give the clam sauce a strong umami flavor [29]. Therefore, sauce III had an umami amino acid content of 1150.79 mg/100 g, which was significantly higher than that of sauces I, II, and IV (996.97 mg/100 g, 900.70 mg/100 g, and 915.46 mg/100 g, respectively). This means that sauce III rendered a superior umami flavor. The bitter amino acid content of sauce III was lower than that of clam sauces I, II, and IV. Among bitter amino acids, Met has a strong bitter taste, and bitter amino acids can produce bitter substances. In summary, the amino acid content of sauce III suggests that it has a better overall flavor than the other sauces, which is also consistent with the observed levels of amino acid nitrogen.

### 3.4. Electronic Nose Analysis of Clam Sauce Favor Profiles

**Figure 3:**
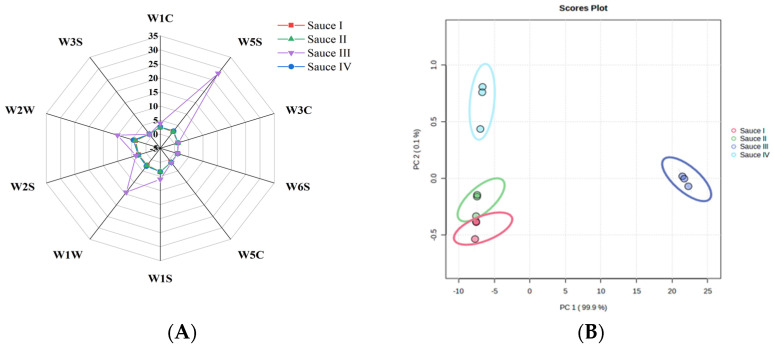
Flavor intensity and principal component analysis of the sauces prepared using different fermentation methods. (**A**) Radar map of the electronic nose. (**B**) Principal component analysis map of the flavor intensity of sauce I, sauce II, sauce III, and sauce IV.

Flavor is an important quality of clam sauce condiments [30]. The flavor profile diagram of the response of 10 sensors in the PEN3 electronic nose to the four different clam sauces is shown in Figure 3A. The flavor profiles of sauce I, sauce II, and sauce IV are essentially the same. The response values of W5S, W1W, and W2W of sauce III were significantly high, indicating that sauce III contained more nitrogen oxides, sulfides, organic sulfides, and aromatic components than sauces I, II, and IV [31]. To further identify the differences among the flavor profiles, the data from the electronic nose were analyzed by principal component analysis (PCA), which clearly showed an obvious distinction between sauces III and I, and between sauces II and IV in PC1. Sauces I, II, and IV could not be clearly distinguished in PC1, indicating the similarity of the flavor profiles of the clam sauces prepared using techniques I, II, and IV. 

### 3.5. Identification of Volatile Compounds in Clam Sauces via HS-SPME/GC-MS

**Figure 4:**
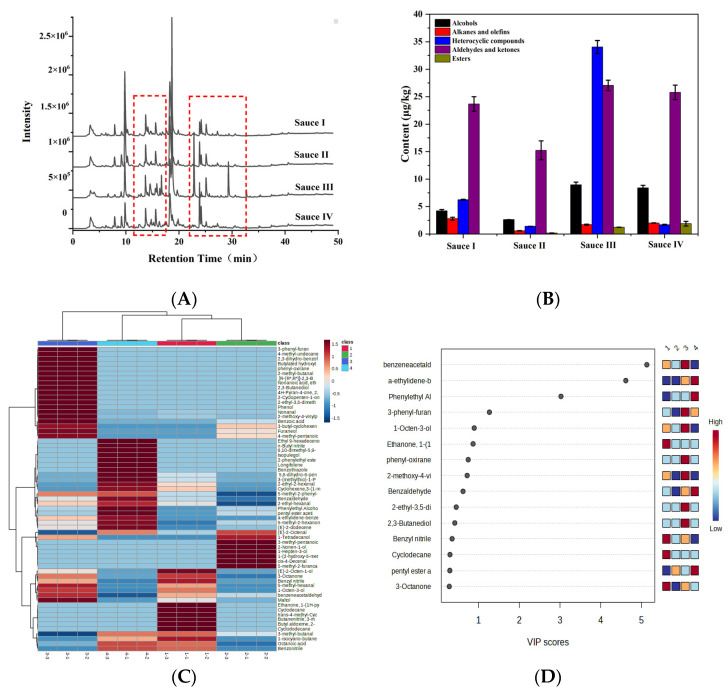
GC-MS and PLS-DA analyses of clam sauces prepared using different fermentation methods. (**A**) GC-MS total ion chromatograms of the clam sauces. (**B**) The total volatile compounds in clam sauces prepared using different fermentation methods. (**C**) Heatmaps of volatile matter spectra in the whole samples prepared with different fermentation methods. (**D**) PLS-DA analysis of the volatile compound content in the whole samples prepared using different fermentation methods (sauce I, sauce II, sauce III, and sauce IV).

To further confirm the difference in volatile flavor compounds in clam sauces fermented using different methods, the clam sauces were analyzed using HS-SPME-GC-MS [32]. Figure 4A shows the chromatogram of the clam sauce samples. Sixty-four volatile compounds, including 22 aldehydes and ketones, 11 alcohols, 13 heterocyclic compounds, five esters, nine alkanes and alkenes, and four acids, were identified and classified into five groups according to their general properties and chemical structures (Table 3).

Aldehydes are among the most important flavor volatiles in clam sauce. They have a low odor threshold and may be derived from the degradation of amino acids or oxidation of unsaturated fatty acids [33]. Aldehydes are therefore important in determining the flavor characteristics of fermented aquatic products. Further, 2-methylbutanal was detected only in sauce III, with a content of 0.83 µg/kg, because of the high fat content of its raw clam meat [34]. Relatively high amounts of phenylacetaldehyde, benzaldehyde, 5-methyl-2-phenyl-2-hexenal, and nonanal were detected in sauces I, II, III, and IV. Benzaldehyde is known to produce sweet odors in foods, including almonds and caramel, and it is one of the most important compounds in the aroma of fermented sauces [35]. Phenacetaldehyde has a strong floral odor [24] and it was found at the highest concentration in sauce III (22.56 µg/kg). These compounds are important components of the flavor of clam sauce and give the sauce a fresh aroma. Additionally, 5-methyl-2-phenyl-2-hexenal can give clam sauce toasty, sweet, and nutty aromas [36]. The contents of nonanal in sauces I, II, III, and IV were 1.54, 0.43, 0.36, and 0.71 µg/kg, respectively, with III having the lowest nonanal content. Nonanals mainly originate from the oxidative decomposition of unsaturated fatty acids, such as linoleic and linolenic acids, and they may also be formed by the Strecker degradation of amino acids [37]. Nonanal can produce an off flavor, which may be responsible for the fishy flavor of clam sauce.

The ketones in clam sauces are produced through the oxidation of unsaturated fatty acids, thermal degradation of amino acids, Maillard reaction, or microbial oxidation [38]. Ketones contribute less to the flavor of clam sauce than aldehydes, owing to their lower relative content having only a slight effect on odor [39]. The differences in the flavors of the various raw materials are mainly due to the difference in the quality and quantity of carbonyl compounds. A total of nine ketones were detected in sauces I, II, III, and IV, with 3-octanone being the most common compound, which can, in turn, give the sauce an odor of mushrooms and butter [39].

Alcohols, which have a botanical and aromatic odor, are derived mainly from the thermal oxidation of lipids and degradation of carbohydrates [38]. In clam sauce, the contribution of alcohols to the aroma is less important than that of aldehydes and ketones, but alcohols still play a key role in the formation of flavor of the clam sauce [40]. Alcohols mainly originate from the degradation of polyunsaturated fatty acids, and 1-octen-3-ol and 2,3 butanediol were the most common alcohols detected in the samples. In addition, 2,3 butanediol, which results in a burnt flavor [41], was detected only in sauce III, with a high relative content of 3.19 µg/kg. This sauce had a particularly strong odor, which demonstrates the importance of this substance for the flavor of sauce III. Another key aroma substance in clam sauce is 1-octene-3-ol, and the relative contents detected in sauces I, II, and III were 2.84, 0.2, and 3.58 µg/kg, respectively, among which sauce III had the highest relative content, giving a mushroom flavor to the overall aroma of the clam sauce [42].

Thirteen heterocyclic compounds were detected in the clam sauces prepared using sauces I, II, III, and IV. Furan is known to greatly enhance the aroma of fermented foods, and it is typically formed via the Amadori rearrangement pathway [35]. The compound 2-ethylfuran has a strong influence on the aroma of clam sauce, because it generates a rubbery, pungent smell in the sauce; therefore, the threshold is low. Moreover, 2-methoxy-4-vinylphenol was detected in sauces I and III, with sauce III having a higher relative content, and 2-methoxy-4-vinylphenol has a typical soy sauce flavor and smokiness and is one of the main components of soy sauce aroma [33]. This molecule is one of the representative compounds that gives clam sauce its characteristic flavor. Pyrazine compounds are the fat oxidation products of the Maillard reaction, which are also characteristic of clam sauce and mainly reflect the flavor of the roast and meat. Alongside that, 2-ethyl-3,5-dimethylpyrazine has a unique soy sauce flavor, and it may be a flavor substance in clam sauce; however, this substance was only detected in sauce III, at a relative content of 3.66 µg/kg. We therefore inferred that this substance could be responsible for the special flavor of sauce III, which differs from those of sauces I, II, and IV. Maltol was also detected, which enriches the clam sauce with a caramel flavor [43].

Esters, produced by the dehydration of hydroxyl fatty acids, usually exhibit a sweet, fruity taste and constitute some portion of the odor component of clam sauce. Five lipids were detected in sauces I, II, III, and IV. In particular, acetic acid, pentyl ester, and nonanoic acid ethyl ester were detected in sauce III, but these lipids may not have strong effects on the aroma of the clam sauce samples [44].

Fourteen hydrocarbons, which are generally aromatic and sweet, were detected in the clam sauce and contributed to its overall flavor to some degree. Alkanes have a weaker effect on odor than other compounds, owing to their higher odor threshold. Alkenes tend to have low odor thresholds, and have a seeming floral and fruity aroma. The clam sauce contained mainly long-chain aliphatic hydrocarbons [29]. Hydrocarbons with a range of 6–19 carbon atoms were detected in the volatiles of crustaceans and fish, but higher thresholds made little contribution to the overall flavor.

The volatile compounds detected in the clam sauce mainly consist of volatile carbonyl compounds and alcohols. Carbonyl compounds, including aldehydes and ketones, are important in the characterization of the product’s odor, owing to their low thresholds. Clam sauce typically contains more aldehydes and ketones than heterocyclic compounds and alcohols (Figure 4B). The total relative content of volatile substances in sauce III was higher than that in sauces I, II, and IV, along with a higher total relative content of alcohols and esters. This demonstrates the superior flavor of clam sauce III.

#### PLS-DA Analysis of Volatile Compound Content in Clam Sauces

A heatmap indicates the differences in the amount of various volatile organic compounds by plotting different shades of colors based on the average amount, which provides an intuitive visualization of the differences between the samples. The relative content of each volatile flavor compound is color-coded in the heat map. The darker the red, the greater the relative content, and the darker the blue, the lower the relative content. As shown in Figure 4C, sauce III contained significantly higher relative levels of nonanal, 2-methoxy-4-vinylphenol, maltol, 1-octene-3-ol, 2-ethyl-3,5-dimethylpyrazine, 2-methylbutaldehyde, 3-octenone, 3-phenylfuran, and phenylacetaldehyde than the other sauces, along with a higher relative content of other volatile compounds, giving it a richer aroma. PLS-DA analysis of the volatile components of clam sauce prepared using different processes was performed based on the obtained heatmaps. The variable importance in projection (VIP) > 1.0 was used to identify whether a substance was an important differential volatile compound. Figure 4D shows four key volatile compounds, phenylacetaldehyde, α-methylene phenylacetaldehyde, phenylethyl alcohol, and 3-phenylfuran (VIP > 1.0), which were found at a higher relative content in sauce III. These results demonstrate the superior flavor quality of sauce III, and they are also consistent with the observations made using the electronic nose.

## 4. Conclusions

The clam sauces prepared using four different fermentation methods (sauce I, sauce II, sauce III, and sauce IV) were analyzed via HS-SPME-GC-MS, which identified 64 volatile compounds. Sauce III, which was prepared through the mixed fermentation of bean koji and clam meat, had the highest free amino acid content of the four sauces, and was superior in flavor. Analysis of the sauces using an electronic nose and tongue demonstrated the superior flavor quality of sauce III and was in good agreement with the GC-MS analysis.

## Figures and Tables

**Table 1:** Sensor array of the E-nose.

MOS	General Description	Sensitive Gas
W1C	Aromatic compounds	Toluene, 10 ppm
W5S	Very sensitive to nitrogen oxides	NO_2_, 1 ppm
W3C	Ammonia, used as a sensor for aromatic compounds	Benzene, 10 ppm
W6S	Mainly hydrogen, selectively (breath gases)	H_2_, 100 ppb
W5S	Alkenes, aromatic compounds, less polar compounds	Propane, 1 ppm
W1S	Sensitive to methane broad range	CH_3_, 100 ppm
W1W	Reacts to sulphur compounds	H_2_S, 1 ppm
W2S	Detects alcohols, partially aromatic compounds	CO, 100 ppm
W2W	Aromatics compounds, sulphur organic compounds	H_2_S, 1 ppm
W3S	Reacts to high concentrations	CH_3_, 100 ppm

**Table 2:** Amino acid analysis of clam sauces ^a^.

Taste	Item	Sauce I	Sauce II	Sauce III	Sauce IV
		FAA ^b^ (mg/100 g)	FAA (mg/100 g)	FAA (mg/100 g)	FAA (mg/100 g)
Umami (+)	Asp	274.90 ± 1.80 ^C^	233.67 ± 3.37 ^B^	299.63 ± 19.03 ^C^	177.73 ± 3.00 ^A^
	Glu	268.37 ± 0.59 ^A^	266.47 ± 3.66 ^A^	330.7 ± 5.30 ^C^	296.53 ± 3.60 ^B^
	Ala	312.77 ± 4.23 ^B^	281.63 ± 2.59 ^A^	328.53 ± 2.21 ^C^	306.13 ± 2.90 ^B^
	Gly	140.93 ± 0.40 ^C^	118.93 ± 1.55 ^A^	191.93 ± 2.15 ^D^	135.07 ± 3.21 ^B^
Total		996.97 ± 5.52	900.7 ± 9.61	1150.79 ± 26.10	915.46 ± 10.57
Sweetness (+)	Ser	168.27 ± 0.81 ^C^	144.87 ± 2.05 ^B^	121.37 ± 3.62 ^A^	158.97 ± 5.77 ^C^
	Thr	119.87 ± 0.35 ^B^	108.33 ± 1.55 ^A^	138.23 ± 6.02 ^C^	164.03 ± 1.96 ^D^
	Pro	93.73 ± 0.46 ^C^	69.53 ± 5.23 ^A^	103.87 ± 2.21 ^D^	85.40 ± 1.91 ^B^
Bitterness/Sweetness (−)	Val	272.77 ± 8.32 ^C^	244.73 ± 18.19 ^B^	179.17 ± 1.58 ^A^	268.5 ± 1.04 ^BC^
	Met	49.3 ± 4.26 ^BC^	42.63 ± 3.21 ^AB^	40.30 ± 0.80 ^A^	55.03 ± 2.37 ^C^
	Lys	106.70 ± 8.66 ^A^	135.70 ± 2.13 ^A^	171.83 ± 1.86 ^B^	140.20 ± 1.39 ^AB^
Bitterness (−)	Ile	90.53 ± 1.37 ^C^	80.30 ± 1.28 ^A^	96.43 ± 1.23 ^C^	78.40 ± 3.46 ^A^
	Leu	170.50 ± 1.87 ^AB^	160.63 ± 2.01 ^A^	171.67 ± 6.41 ^B^	173.63 ± 3.06 ^B^
	Tyr	21.40 ± 3.91 ^B^	19.93 ± 0.40 ^B^	7.30 ± 0.30 ^A^	23.1 ± 0.35 ^B^
	Phe	103.80 ± 9.88 ^A^	108.87 ± 2.44 ^A^	162.73 ± 1.60 ^B^	110.17 ± 4.39 ^A^
	TFAA ^c^	2193.84 ± 17.52	2016.22 ± 15.43	2343.69 ± 40.19	2172.89 ± 23.14

^a^ Mean value ± standard deviation (*n* = 3). ^b^ Means delicious amino acids (FAAs). ^c^ Total free amino acids (TFAAs). A–D different superscripts in the same row indicate significant differences (*p* < 0.05).

**Table 3:** Volatile compounds in the sauce identified via HS-SPME-GC-MS.

Compounds ^d^	Molecular Formula	CAS	MV	Characterization	RI ^a^	RI ^b^	RT (min)	Sauce I	Sauce II	Sauce III	Sauce IV
								^c^ Content	Content	Content	Content
Alcohols (11)											
Phenylethyl alcohol	C_8_H_10_O	60-12-8	122	91	1906	1889	22.77	0.46 ± 0.02	1.76 ± 0.02	ND	7.30 ± 0.47
(E)-2-Octen-1-ol	C_8_H_16_O	18409-17-1	128	57	1614	1595	18.25	0.59 ± 0.29	0.38 ± 0.01	0.16 ± 0.02	ND
1-Octen-3-ol	C_8_H_16_O	3391-86-4	128	57	1450	1458	13.69	2.84 ± 0.29	0.20 ± 0.00	3.58 ± 0.18	ND
3-(methylthio)-1-propanol	C_4_H_10_OS	505-10-2	106	61	1719	1709	19.84	0.16 ± 0.03	ND	0.22 ± 0.01	0.79 ± 0.01
1-Tetradecanol	C_20_H_42_O	629-96-9	214	55	2171	2165	29.03	ND	0.09 ± 0.00	0.06 ± 0.00	ND
Trans-4-methyl-cyclohexanol	C_7_H_14_O	7731-29-5	114	57	1452	1444	14.49	0.16 ± 0.02	ND	ND	ND
1-Hepten-3-ol	C_7_H_14_O	4938-52-7	114	57	1351	1332	11.86	ND	0.03 ± 0.00	ND	ND
2-Nonen-1-ol	C_9_H_18_O	22104-79-6	142	57	1692	-	5.51	ND	0.19 ± 0.03	ND	ND
2,3-Butanediol	C_4_H_10_O_2_	107-88-0	90	57	1543	1542	16.69	ND	ND	3.19 ± 0.26	ND
[R-(R*,R*)]-2,3-butanediol	C_4_H_10_O_2_	24347-58-8	90	57	-	1502	15.81	ND	ND	1.95 ± 0.05	ND
Isopulegol	C_10_H_18_O	89-79-2	154	68	1571	1554	16.93	ND	ND	ND	0.33 ± 0.03
Alkanes and Olefins (9)											
(E)-2-dodecene	C_12_H_24_	7206-13-5	168	55	1265	1260	10.19	ND	0.11 ± 0.08	0.23 ± 0.01	0.58 ± 0.02
3-(1-methylpropyl)-cyclohexene	C_10_H_18_	15232-91-4	138	81	1187	1186	8.55	0.20 ± 0.12	ND	ND	0.36 ± 0.01
1-isocyano-butane	C_5_H_9_N	2769-64-4	83	55	-	-	5.51	0.59 ± 0.04	ND	ND	0.40 ± 0.04
3-methyl-butanenitrile	C_5_H_9_N	625-28-5	83	68	1129	-	6.18	0.22 ± 0.04	ND	ND	ND
Cyclodecane	C_10_H_20_	293-96-9	140	55	1261	1261	10.23	0.81 ± 0.25	ND	ND	ND
Butyl aldoxime, 2-methyl-, anti-	C_5_H_11_NO	49805-55-2	101	56	1486	1468	15.55	0.25 ± 0.19	ND	ND	ND
Cyclododecane	C_12_H_24_	294-62-2	168	55	1517	1526	17.36	0.56 ± 0.40	ND	ND	ND
4-methyl-undecane	C_12_H_26_	2980-69-0	170	71	1147	1160	7.87	ND	ND	0.58 ± 0.03	ND
Longifolene	C_15_H_24_	475-20-7	204	161	1577	1566	17.20	ND	ND	ND	0.23 ± 0.03
Aldehydes and Ketones (22)											
Nonanal	C_9_H_18_O	124-19-6	142	57	1391	1377	12.54	1.54 ± 0.14	0.43 ± 0.00	0.36 ± 0.01	0.71 ± 0.05
3-Octanone	C_8_H_16_O	106-68-3	128	57	1253	1236	9.12	2.05 ± 0.29	0.72 ± 0.04	0.90 ± 0.04	1.71 ± 0.03
Benzaldehyde	C_7_H_6_O	100-52-7	106	106	1520	1502	16.84	1.50 ± 0.69	1.46 ± 0.00	1.08 ± 0.10	3.11 ± 0.02
Benzeneacetaldehyde	C_10_H_10_O	503-74-2	129	91	1640	1633	18.70	16.13 ± 1.59	9.57 ± 1.76	22.56 ± 0.73	14.95 ± 0.57
2-ethyl-hexanal	C_8_H_16_O	123-05-7	128	72	1213	1213	8.93	0.07 ± 0.02	0.03 ± 0.01	0.04 ± 0.01	0.08 ± 0.01
5-methyl-2-phenyl-2-hexenal	C_13_H_16_O	21834-92-4	188	117	2056	2068	27.21	0.30 ± 0.05	0.31 ± 0.13	0.44 ± 0.00	0.48 ± 0.02
(E)-2-Octenal	C_8_H_14_O	2548-87-0	126	55	1429	1444	13.45	0.38 ± 0.29	0.38 ± 0.01	ND	0.35 ± 0.01
3-methyl-butanal	C_5_H_10_O	590-86-3	86	58	918	-	3.29	2.33 ± 0.07	ND	ND	2.61 ± 0.57
2-ethyl-2-hexenal	C_8_H_14_O	645-62-5	126	55	1333	1331	11.86	0.10 ± 0.05	ND	ND	0.16 ± 0.02
5-methyl-hexanal	C_7_H_14_O	1860-39-5	114	58	1150	1145	7.53	0.20 ± 0.12	ND	0.16 ± 0.01	ND
5,6-dihydro-6-pentyl-2H-Pyran-2-one	C_10_H_16_O_2_	54814-64-1	168	97	2227	2238	30.28	0.05 ± 0.02	ND	ND	0.17 ± 0.01
Furaneol	C_6_H_8_O_3_	3658-77-3	128	57	2031	2016	26.29	ND	0.07 ± 0.00	0.17 ± 0.01	ND
5-methyl-2-hexanone	C_7_H_14_O	110-12-3	114	58	1156	1147	7.56	ND	0.12 ± 0.00	0.16 ± 0.01	0.36 ± 0.02
3-methyl-butanal	C_5_H_10_O	590-86-3	86	58	918	-	3.32	ND	ND	ND	1.37 ± 0.04
Cis-4-decenal	C_10_H_18_O	21662-09-9	154	55	1544	1529	16.40	ND	0.42 ± 0.02	ND	ND
5-methyl-2-furancarboxaldehyde	C_12_H_24_O	118447-56-6	110	110	1570	1554	16.93	ND	0.16 ± 0.01	ND	ND
1-(2-hydroxy-5-methylphenyl)-ethanone	C_9_H_10_O_2_	1450-72-2	150	135	2185	2187	29.40	ND	0.07 ± 0.01	ND	ND
2-methyl-butanal	C_5_H_10_O	96-17-3	86	58	914	-	3.32	ND	1.37 ± 0.04	0.83 ± 0.07	ND
2-cyclopenten-1-one	C_6_H_8_O_2_	80-71-7	112	112	1830	1812	22.36	ND	ND	0.08 ± 0.00	ND
2,3-dihydro-3,5-dihydroxy-6-methyl-4H-Pyran-4-one,	C_6_H_8_O_4_	28564-83-2	144	144	2267	2249	30.43	ND	ND	0.18 ± 0.04	ND
α-ethylidene-benzeneacetaldehyde	C_10_H_10_O	503-74-2	129	91	1640	1620	18.31	ND	ND	5.36 ± 0.28	ND
6,10-dimethyl-5,9-undecadien-2-one	C_13_H_22_O	689-67-8	194	69	1845	1841	23.03	ND	ND	ND	0.25 ± 0.06
heterocyclic compounds (13)											
3-phenyl-furan	C_10_H_8_O	13679-41-9	144	144	1849	1836	21.94	0.18 ± 0.04	0.11 ± 0.00	8.19 ± 0.31	0.21 ± 0.02
Benzyl nitrile	C_8_H_7_N	140-29-4	117	117	1910	1905	23.04	1.02 ± 0.07	0.16 ± 0.01	0.70 ± 0.04	0.08 ± 0.02
Maltol	C_6_H_6_O_3_	118-71-8	126	126	1969	1952	24.16	1.99 ± 0.26	1.14 ± 0.02	4.13 ± 0.17	0.90 ± 0.03
2-methoxy-4-vinylphenol	C_9_H_10_O_2_	7786-61-0	150	107	2188	2182	29.38	0.38 ± 0.08	ND	5.64 ± 0.19	ND
2-pentyl-furan	C_10_H_18_	3983-7-1	138	81	1231	1184	8.45	ND	0.09 ± 0.00	0.16 ± 0.02	ND
Benzonitrile	C_7_H_5_N	100-47-0	103	103	1585	1574	17.64	0.33±	ND	ND	0.35 ± 0.06
1-(1H-pyrrol-2-yl)-ethanone	C_6_H_7_NO	1072-83-9	109	94	1973	1953	25.09	2.33 ± 0.77	ND	ND	ND
2-ethyl-3,5-dimethyl-pyrazine	C_8_H_12_N_2_	1124-11-4	136	135	1455	1447	14.57	ND	ND	3.66 ± 0.16	ND
Phenyl-oxirane	C_8_H_8_O	122-78-1	120	91	1631	1616	18.53	ND	ND	6.03 ± 0.95	ND
Butylated hydroxytoluene	C_15_H_24_O	128-37-0	220	205	1909	1890	23.91	ND	ND	0.35 ± 0.01	ND
Phenol	C_6_H_6_O	108-95-2	94	94	2000	1984	25.71	ND	ND	0.39 ± 0.01	ND
2,3-dihydro-benzofuran	C_8_H_8_O	496-16-2	120	120	2389	2383	32.69	ND	ND	1.13 ± 0.10	ND
Benzothiazole	C_7_H_5_NS	95-16-9	135	135	1958	1945	24.96	ND	ND	ND	0.15 ± 0.06
Esters (5)											
Pentyl ester acetic acid	C_7_H_14_O_2_	628-63-7	130	70	1176	-	6.21	ND	0.17 ± 0.02	0.13 ± 0.02	0.68 ± 0.03
Nonanoic acid, ethyl ester	C_4_H_8_O_2_	79-31-2	186	88	1531	1527	16.37	ND	ND	1.11 ± 0.00	ND
n-Butyl nitrite	C_4_H_9_NO_2_	544-16-1	103	60	-	1409	13.70	ND	ND	ND	0.52 ± 0.43
2-phenylethyl ester acetic acid	C_10_H_12_O_2_	103-45-7	164	104	1813	1796	21.98	ND	ND	ND	0.62 ± 0.01
Ethyl 9-hexadecenoate	C_18_H_34_O_2_	54546	281	55	2281	2279	31.00	ND	ND	ND	0.07 ± 0.01
Acids(4)											
Octanoic acid	C_8_H_16_O_2_	124-07-2	144	60	2060	2044	26.82	0.19 ± 0.05	0.07 ± 0.02	0.10 ± 0.01	0.20 ± 0.00
4-methyl-pentanoic acid	C_6_H_12_O_2_	646-07-1	116	57	1803	1820	22.56	ND	0.21 ± 0.02	0.43 ± 0.03	ND
3-methyl-pentanoic acid	C_6_H_12_O_2_	105-43-1	116	60	1782	1796	22.05	ND	0.10 ± 0.1	ND	ND
Benzoic acid	C_9_H_12_O_2_	80-15-9	122	105	2412	2425	33.37	ND	0.05 ± 0.00	0.22 ± 0.00	ND

^a^ RI is derived from the NIST mass spectrometry database. ^b^ Retention index was calculated by the interpolation of the retention times of a C8–C30 n-alkanes series under the same chromatographic conditions. ^c^ Data are represented as the mean ± SD. ^d^ Identification via GC/MS. ND: volatile flavor compounds not detected.

## Data Availability

Data are contained within the article.
